# Tubulointerstitial nephritis antigen‐like 1 deficiency alleviates age‐dependent depressed ovulation associated with ovarian collagen deposition in mice

**DOI:** 10.1002/rmb2.12301

**Published:** 2019-09-21

**Authors:** Masato Akaiwa, Emiko Fukui, Hiromichi Matsumoto

**Affiliations:** ^1^ Laboratory of Animal Breeding and Reproduction Division of Animal Science School of Agriculture Utsunomiya University Utsunomiya Japan; ^2^ Center for Bioscience Research and Education Utsunomiya University Utsunomiya Japan

**Keywords:** aging, matricellular protein, mouse, ovulation, Tinagl1

## Abstract

**Purpose:**

This study aimed to examine whether the Tinagl1 might be associated with ovulation in aged females and reproductive age‐associated fibrosis in the stroma of the ovary.

**Methods:**

To address the ovulatory ability and quality of ovulated oocytes, we induced ovulation by treatment with equine chorionic gonadotropin (eCG) and human chorionic gonadotropin (hCG) followed by in vitro fertilization. We also performed Picrosirius Red (PSR) staining to evaluate ovarian collagen deposition.

**Results:**

As compared to ovulation in 8‐ to 9‐month‐old *Tinagl1^flox/flox^* mice, the number of ovulated oocytes from *Tinagl1^flox/flox^* mice decreased in an age‐dependent manner in mice more than 10‐11 months old, whereas the ovulated oocyte numbers in *Tinagl1* ^−/−^ mice decreased significantly at 14‐15 months. In vitro fertilization followed by embryo culture demonstrated the normal developmental potential of *Tinagl1*‐null embryos during the preimplantation period. PSR staining indicated that collagen was found throughout the ovarian stroma in an age‐dependent manner in *Tinagl1^flox/flox^* females, whereas those distributions were delayed to 14‐15 months in *Tinagl1* ^−/−^ females. This timing was consistent with the delayed timing of age‐related decline of ovulation in *Tinagl1* ^−/−^ females.

**Conclusions:**

The alleviation of age‐associated depression of ovulation was caused by delayed ovarian collagen deposition in *Tinagl1*‐null female mice.

## Graphical abstract

Effect of female aging on numbers of ovulated oocytes fromTinagl1‐deficient mice.

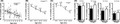

## 1 INTRODUCTION

In mammals, advanced maternal physiologic age and fertility are inversely correlated.^1^, ^2^ Age‐associated reproductive decline is multifactorial and is largely due to a constant reduction in the number of available oocytes.^3^, ^4^, ^5^ Moreover, reduced mammalian fertility is also influenced by oocyte quality and an oocyte's developmental potential; decreased ooplasm quality, mitochondrial defects, and abnormalities in the meiotic machinery are all potential contributors to the age‐related decline in oocyte quality observed in mice and humans.^1^, ^2^, ^6^ Indeed, developmental potential can be improved after transfer of oocyte genomes from aged mice into oocytes of young mice.^7^, ^8^

In addition, the reduction of gamete quantity is characterized as physiologic aging. While the number of available oocytes is reduced in an age‐dependent manner, rare follicles were observed in ovaries, and the ovarian cortex was almost displaced by connective tissue in aged mice.^1^ In mice, reproductive age‐associated fibrosis occurred in the stroma of the ovary, as evidenced by the observation that Picrosirius Red (PSR) staining, specific for collagen I and III, was minimal in ovaries from reproductively young adult mice (6‐12 weeks), increased in distinct foci in mice of mid‐to‐advanced reproductive age, and prominent throughout the stroma of the oldest mice in the study (>20 months).^9^ Therefore, the distribution of extracellular matrix (ECM) components appears to be involved in age‐associated reproductive decline, including ovulation.

In contrast to the ECM proteins such as collagens, which contribute to the structural integrity of the extracellular matrix by multimerization, a group of ECM proteins that do not directly play a structural role is categorized as matricellular proteins.^10^ Matricellular proteins interact with structural matrix proteins, cell surface receptors, or extracellular factors such as growth factors and proteinases, and modulate cell‐matrix interactions and cellular functions, including proliferation, differentiation, adhesion, and migration. Furthermore, several matricellular proteins that are associated with collagen deposition are known to be involved in hepatic fibrosis, including osteopontin (OPN),^11^, ^12^, ^13^ tenascin C,^14^ cysteine‐rich acidic secreted protein (SPARC),^15^, ^16^, ^17^ and periostin.^18^

Tubulointerstitial nephritis antigen‐like 1 (Tinagl1, also known as adrenocortical zonation factor 1 [AZ‐1] or lipocalin 7) is a matricellular protein that interacts with both structural matrix proteins and cell surface receptors.^19^, ^20^, ^21^ Our previous study revealed that homologous mating of *Tinagl1^−/−^* females and *Tinagl1* ^−/−^ males showed impaired fertility during pregnancy, including failure to carry pregnancy to term and perinatal lethality.^22^ Meanwhile, ovulation from 2‐ to 7‐month‐old *Tinagl1^−/−^* mice induced by equine chorionic gonadotropin (eCG) and human chorionic gonadotropin (hCG) showed that the number of ovulated oocytes did not differ compared with the number in *Tinagl1^flox/flox^* mice.^22^ In vitro fertilization followed by embryo culture also demonstrated normal developmental potential of *Tinagl1*‐null embryos during the preimplantation period.^22^

In aged mice, the ovarian cortex was almost displaced by connective tissue.^1^ Furthermore, reproductive age‐associated fibrosis was apparent in the stroma of the ovary in mice.^9^ As Tinagl1 is a matricellular protein, *Tinagl1* deficiency could be associated with ovulation in aged females. Our initial expectation in the present study was that age‐associated depression of ovulation in aged *Tinagl1^−/−^* females might be slight as compared with *Tinagl1^flox/flox^* females. To address the ovulatory ability and quality of ovulated oocytes, we induced ovulation by treatment with eCG and hCG followed by in vitro fertilization. We also performed PSR staining in ovaries to examine the accumulation of collagen and investigated whether the amount of collagen deposition differed between *Tinagl1* ^−/−^ and *Tinagl1^flox/flox^* during aging.

## 2 MATERIALS AND METHODS

### 2.1 Animals

*Tinagl1*‐deficient mice were generated as described previously.^22^ *Tinagl1* ^−/−^ and *Tinagl1^flox/flox^* mice were maintained by homologous matings. These mice were in the C57BL/6NCr background. Genotype was determined by PCR analysis of genomic DNA. Mice were bred in our animal care facility. The animal experiments described here were approved by the Animal Experimentation Committee at the Utsunomiya University and were performed in accordance with the instructions in the Guide for the Care and Use of Laboratory Animals published by Utsunomiya University.

### 2.2 Superovulation, in vitro fertilization, and embryo culture

Superovulation, in vitro fertilization, and culture of embryos were performed as previously described.^22^, ^23^ Female mice were subjected to superovulation by intraperitoneal injection of 5 IU eCG (ASKA Animal Health Co., Ltd.) followed by 5 IU hCG (ASKA Animal Health Co., Ltd.) 48 hours later. Ovulated oocytes from *Tinagl1* ^−/−^ or *Tinagl1^flox/flox^* females were then collected in human tubal fluid (HTF) medium without phenol red (HTF‐P) 14 hours after hCG injection. Spermatozoa were obtained from *Tinagl1* ^−/−^ or *Tinagl1^flox/flox^* males and preincubated for 2‐3 hours in HTF‐P to allow for capacitation; the final concentration was 700 spermatozoa/µL. In vitro fertilization was performed with homozygous combinations of *Tinagl1* ^−/−^ or *Tinagl1^flox/flox^* for oocytes and spermatozoa. Four hours after insemination, the oocytes were transferred into 100 µL of potassium simplex optimized medium (KSOM) without phenol red (KSOM‐P), overlaid with paraffin liquid (Nacalai Tesque) and cultured in a humidified atmosphere with 5% CO_2_ at 37°C.

### 2.3 Picrosirius Red (PSR) staining

Picrosirius Red staining was performed as previously described^9^, ^24^, ^25^ with slight modifications. Ovarian tissues were snap frozen following collection of ovulated oocytes using freezing solution (FREEZER, HOZAN) as described previously.^20^, ^21^, ^26^ Frozen 10‐µm sections obtained using a cryostat were mounted onto silane‐coated glass slides (Matsunami Glass Ind., Ltd.). Sections were fixed in cold acetone on ice for 10 minutes followed by washing in PBS. Slides were then immersed in a PSR staining solution prepared by dissolving Sirius Red (FUJIFILM Wako Pure Chemical Co.) in a saturated aqueous solution of picric acid (FUJIFILM Wako Pure Chemical Co.) at 0.03% w/v for 10 minutes at 23‐26°C. The stained sections were then washed for 2 minutes in 0.01 N HCl, dehydrated in 100% ethanol (a total of three, 30 seconds incubations), cleared in xylene for 5 minutes, and mounted with mounting medium (Daido Sangyo Co.). ImageJ software (National Institutes of Health) was used to process color threshold images of PSR‐stained ovarian tissue sections as described previously.^9^ Three mice were used for each genotype and age group.

### 2.4 Immunohistochemical staining of Tinagl1

Immunohistochemistry was performed as described previously.^20^, ^21^, ^22^ Frozen 10‐µm sections from snap‐frozen tissues were mounted onto silane‐coated glass slides. Sections were fixed in cold acetone on ice, immersed in 3% hydrogen peroxide in methanol, and treated with 1% BSA in PBS. For the primary antibody reaction, sections were incubated with rabbit polyclonal antibody to Tinagl1. After washing, sections were incubated with biotinylated goat anti‐rabbit antibody (Thermo Fisher Scientific, Inc). After incubation with horseradish peroxidase‐conjugated streptavidin (Thermo Fisher Scientific, Inc), reactions were visualized using 3‐amino‐9‐ethyl carbazole (Thermo Fisher Scientific, Inc) as a chromogen, followed by counterstaining with hematoxylin. Reddish deposits indicate the sites of immunoreaction.

### 2.5 Statistical analysis

The relationship between age and fertility was determined by Pearson correlation coefficient and linear regression analysis. One‐way factorial ANOVA followed by Fisher's protected least significant difference test was used to evaluate differences among age groups of the same genotype. Student's *t* test was used to evaluate differences between different genotypes in the same age group. *P* < .05 was considered statistically significant.

## 3 RESULTS

### 3.1 Tinagl1‐deficient female mice exhibit alleviation of age‐related ovulation decline

**Figure 1:**
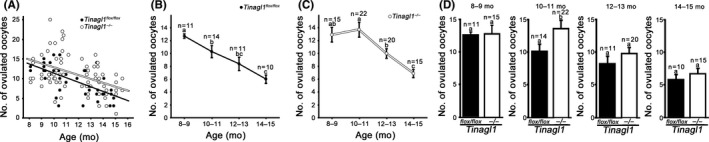
Effect of female aging on numbers of ovulated oocytes from *Tinagl1^flox/flox^* and *Tinagl1^−/−^* mice. A, Scatterplots displaying the correlation between age and numbers of ovulated oocytes. B, Bimonthly profile of the numbers of ovulated oocytes (mean ± SEM) derived from *Tinagl1^flox/flox^* females. Different letters (a–c above error bars) indicate significant differences among the age groups (*P* < .05). C, Bimonthly profile of the numbers of ovulated oocytes derived from *Tinagl1^−/−^* females. Different letters (a–c above error bars) indicate significant differences among the age groups (*P* < .05). D, Comparisons of ovulated oocyte numbers between *Tinagl1^flox/flox^* and *Tinagl1^−/−^* females in matched age groups. Different letters (a and b above error bars) indicate significant differences between *Tinagl1^flox/flox^* and *Tinagl1^−/−^* mice (*P* < .05)

*Tinagl1^flox/flox^* and *Tinagl1^−/−^* females were stimulated with hormones, and superovulated oocytes were collected. A scatterplot depicting the age (8‐15 months old) and the numbers of ovulated oocytes obtained from each of the *Tinagl1^flox/flox^* (n = 46) and *Tinagl1^−/−^* (n = 72) females is shown in Figure 1A. The linear regression formulas for the number of ovulated oocytes (*Y*) and age (*X*) from *Tinagl1^flox/flox^* and *Tinagl1^−/−^* mice were *Y* = −1.16*X* + 23.03 and *Y* = −0.98*X* + 22.74, respectively. The Pearson correlation coefficients (*r*) obtained from *Tinagl1^flox/flox^* and *Tinagl1^−/−^* were −0.64 and −0.44, respectively, (both *P* < .05). The numbers of ovulated oocytes from *Tinagl1^flox/flox^* mice from the different age groups are summarized in Figure 1B. The numbers of ovulated oocytes (mean ± SEM) from 8‐ to 9‐, 10‐ to11‐, 12‐ to 13‐, and 14‐ to 15‐month‐old mice were 12.7 ± 0.4 (n = 11), 10.3 ± 1.0 (n = 14), 8.4 ± 1.1 (n = 11), and 6.0 ± 0.7 (n = 10), respectively. As compared with the 8‐ to 9‐month‐old mice, the numbers of ovulated oocytes decreased in mice more than 10‐11 months old in an age‐dependent manner (*P* < .05). The number of ovulated oocytes from *Tinagl1^−/−^* mice among different age groups is summarized in Figure 1C. The number of ovulated oocytes per 8‐ to 9‐, 10‐ to 11‐, 12‐ to 13‐, and 14‐ to 15‐month‐old mouse was 12.9 ± 1.2 (n = 15), 13.7 ± 1.2 (n = 22), 10.0 ± 0.8 (n = 20), and 6.9 ± 0.7 (n = 15), respectively. As compared with 8‐ to 9‐month‐old mice, the numbers of ovulated oocytes were not significantly different from those that were 10‐11 and 12‐13 months old; however, a significant decrease was observed by 14‐15 months old (*P* < .05). The number of ovulated oocytes in matched age groups from mice of different genotypes is shown in Figure 1D. In the 10‐ to 11‐month‐old group, the number of ovulated oocytes from *Tinagl1^−/−^* mice was higher as compared with *Tinagl1^flox/flox^* mice (*P* < .05). These results indicate that deficiency of *Tinagl1* alleviates the age‐related decline of ovulation.

### 3.2 Cleavage of fertilized eggs is not affected by Tinagl1‐deficiency

**Figure 2:**
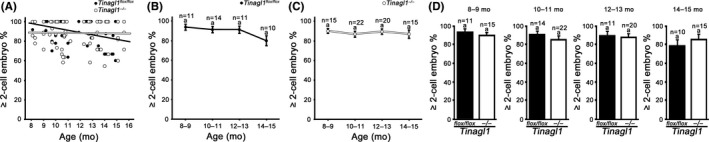
Effect of female aging on cleavage of fertilized eggs derived from *Tinagl1^flox/flox^* and *Tinagl1^−/−^*mice to the 2‐cell stage or beyond. A, Scatterplots displaying the correlation between age and percentages of fertilized eggs. B, Bimonthly profile of the percentages of fertilized eggs derived from *Tinagl1^flox/flox^* females. The same letter (a above error bars) indicates no significant differences among the age groups (*P* > .05). C, Bimonthly profile of the percentages of fertilized eggs derived from *Tinagl1^−/−^* females. The same letter (a above error bars) indicates no significant differences among the age groups (*P* > .05). D, Comparisons of the percentages of fertilized eggs between *Tinagl1^flox/flox^* and *Tinagl1^−/−^* mice in the same age groups. The same letter (a above error bars) indicates no significant differences between *Tinagl1^flox/flox^* and *Tinagl1^−/−^* (*P* > .05)

As described above, age‐related declines in ovulation were alleviated in *Tinagl1*‐deficient female mice. To address the developmental potential of oocytes, in vitro fertilization was employed to determine the competency of ovulated oocytes for fertilization. Cleavage of fertilized eggs to the 2‐cell stage or beyond was used as a parameter for successful in vitro fertilization. The scatterplot in Figure 2A depicts the age (8‐15 months old) and the percentage of cleaved eggs obtained from each of the *Tinagl1^flox/flox^* (n = 46) and *Tinagl1^−/−^* (n = 72) females. Linear regression formulas for the percentage of cleaved eggs (*Y*) and age (*X*) obtained from *Tinagl1^flox/flox^* and *Tinagl1^−/−^* females were *Y* = −2.32*X* + 116.60 and *Y* = −0.13*X* + 89.93, respectively. The Pearson correlation coefficient (*r*) obtained for *Tinagl1^flox/flox^* was −0.36 (*P* < .05). In contrast, the *r* for *Tinagl1^−/−^*, *r* was −0.02, and there was no significant correlation between percentage of cleaved eggs and age (*P* > .05). The percentages of cleaved eggs from *Tinagl1^flox/flox^* mice in different age groups are summarized in Figure 2B. The percentages of cleaved eggs (mean ± SEM) from 8‐ to 9‐, 10‐ to 11‐, 12‐ to 13‐, and 14‐ to 15‐month‐old mice were 93.9 ± 3.1 (n = 11), 91.4 ± 3.1 (n = 14), 91.3 ± 3.7 (n = 11), and 80.0 ± 5.2 (n = 10), respectively. There were no significant differences among these age groups (*P* > .05). Figure 2C shows the percentages of cleaved eggs from *Tinagl1^−/−^* mice in different age groups. The percentages of cleaved eggs (mean ± SEM) from 8‐ to 9‐, 10‐ to 11‐, 12‐ to 13‐, and 14‐ to 15‐month‐old mice were 90.2 ± 2.5 (n = 15), 86.9 ± 3.1 (n = 22), 89.5 ± 2.8 (n = 20), and 87.1 ± 4.3 (n = 15), respectively. There were no significant differences among these groups (*P* > .05). The percentages of cleaved eggs in matched age groups from mice of different genotypes are summarized in Figure 2D. The percentages of cleaved eggs in *Tinagl1^flox/flox^* and *Tinagl1^−/−^* mice were not significantly different for any of the age groups (*P* > .05). These results indicate that the fertilization potential of ovulated oocytes did not differ between *Tinagl1^flox/flox^* and *Tinagl1^−/−^* mice.

### 3.3 Development to blastocyst is not affected by Tinagl1‐deficiency

**Figure 3:**
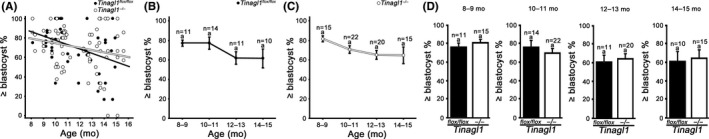
Effect of female aging on development of oocytes derived from *Tinagl1^flox/flox^* and *Tinagl1^−/−^* females to the blastocyst stage or beyond. A, Scatterplots displaying the correlation between age and percentages of blastocysts. B, Bimonthly profile of the percentages of blastocysts derived from *Tinagl1^flox/flox^* females. The same letter (a above error bars) indicates no significant differences among the age groups (*P* > .05). C, Bimonthly profile of the percentages of blastocysts derived from *Tinagl1^−/−^* females. The same letter (a above error bars) indicates no significant differences among the age groups (*P* > .05). D, Comparisons of the percentages of blastocysts between *Tinagl1^flox/flox^* and *Tinagl1^−/−^* in matched age groups. The same letter (a above error bars) indicates no significant differences between *Tinagl1^flox/flox^* and *Tinagl1^−/−^* (*P* > .05)

As described above, the fertilization potential of ovulated oocytes did not differ significantly between *Tinagl1^flox/flox^* and *Tinagl1^‐/ ‐^* mice. To address the further developmental ability, embryo culture was employed to determine the preimplantation development to the blastocyst stage. Development to the blastocyst stage or beyond was used as a parameter for successful in vitro development during the preimplantation period. The scatterplot in Figure 3A shows the age (8‐15 months old) and the percentage of blastocysts obtained from each of the *Tinagl1^flox/flox^* (n = 46) and *Tinagl1^−/−^* (n = 72) genotype females. Linear regression between the percentage of blastocysts (*Y*) and the age (*X*) obtained from *Tinagl1^flox/flox^* and *Tinagl1^−/−^* mice yielded the following formulas: *Y* = −4.43*X* + 121.90 and *Y* = −2.33*X* + 97.82, respectively. The Pearson correlation coefficient (*r*) obtained for *Tinagl1^flox/flox^* females was −0.39 (*P* < .05). In contrast, for *Tinagl1^−/−^* females, *r* was −0.23, and there was no significant correlation between the percentage of blastocysts and age (*P* > .05). The percentages of blastocysts from *Tinagl1^flox/flox^* mice in different age groups are summarized in Figure 3B. The percentages (mean ± SEM) of blastocysts from 8‐ to 9‐, 10‐ to 11‐, 12‐ to 13‐ and 14‐ to 15‐months‐old females were 77.3 ± 3.3 (n = 11), 77.3 ± 6.4 (n = 14), 61.9 ± 6.6 (n = 11), and 61.6 ± 9.8 (n = 10), respectively. The percentages of blastocysts were not significantly different among the age groups (*P* > .05). The percentages of blastocysts from *Tinagl1^−/−^* mice in the different age groups are shown in Figure 3C. The percentages of blastocysts (mean ± SEM) from 8‐ to 9‐, 10‐ to 11‐, 12‐ to 13‐, and 14‐ to 15‐month‐old females were 81.6 ± 3.1 (n = 15), 70.7 ± 4.0 (n = 22), 65.3 ± 4.6 (n = 20), and 64.8 ± 8.6 (n = 15), respectively. There were no significant differences among these groups (*P* > .05). Figure 3D shows the percentages of blastocysts in the same age groups from mice with different genotypes. The percentages of blastocysts did not differ significantly between *Tinagl1^flox/flox^* and *Tinagl1^−/−^* females for any of the age groups (*P* > .05). These results indicate that the developmental potential of ovulated oocytes to the blastocyst stage did not differ between the *Tinagl1^flox/flox^* and the *Tinagl1^−/−^* mice.

### 3.4 Ovarian deposition of collagen is retarded by Tinagl1‐deficiency

**Figure 4:**
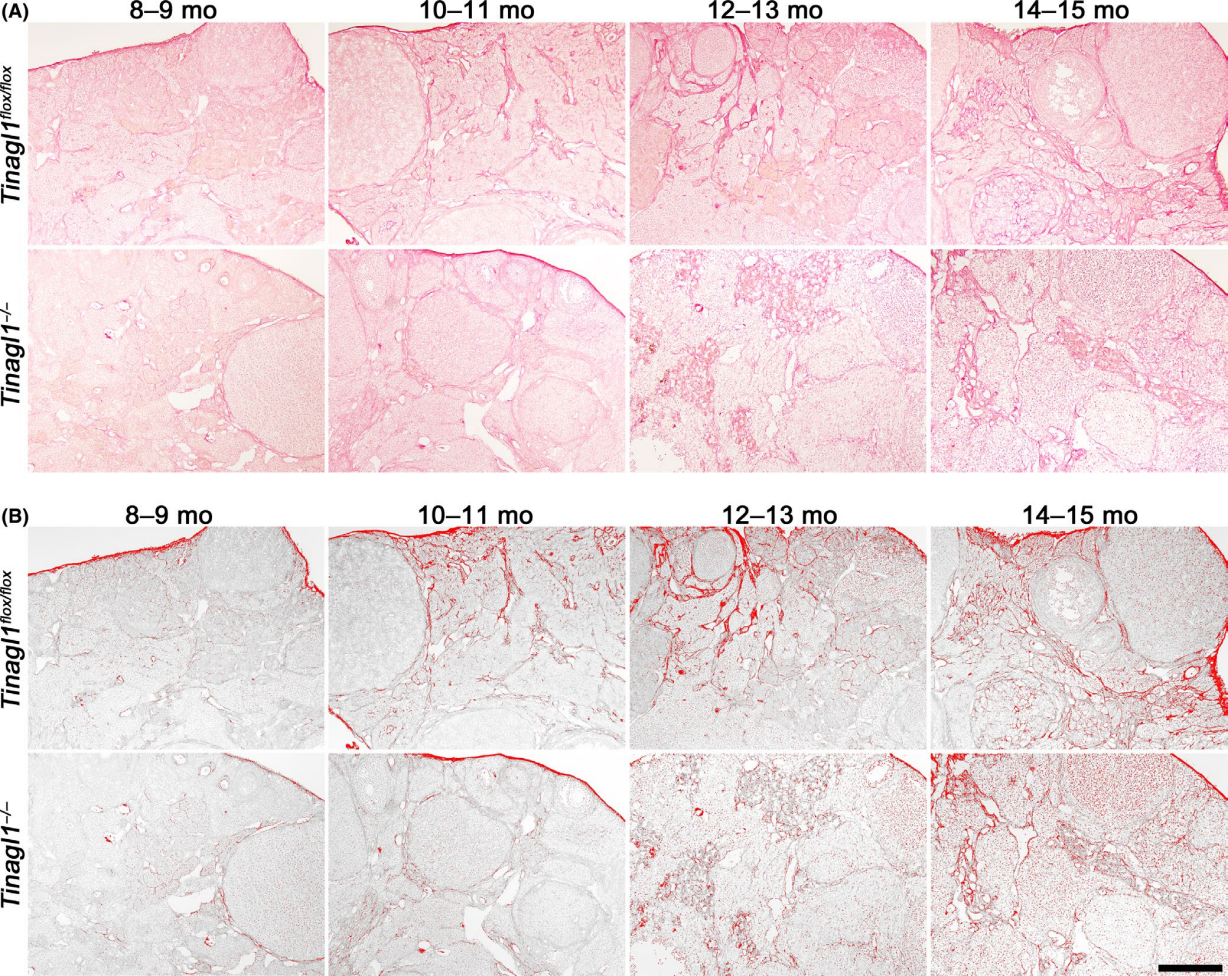
PSR staining and the resulting processed color threshold images. PSR staining in ovaries derived from *Tinagl1^flox/flox^* and *Tinagl1* ^−/−^ females during aging (A). Representative processed color threshold images of PSR‐stained ovarian tissue sections (B). The scale bar represents 300 µm

As shown in Figure 1, *Tinagl1*‐deficient female mice exhibited alleviation of age‐related ovulation decline. Therefore, we hypothesized that reproductive age‐associated deposition of collagen may take place in the stroma of the ovary during aging and that the age‐associated deposition of collagen also may be delayed in *Tinagl1* ^−/−^ as compared with *Tinagl1^flox/flox^* mice. To address this issue, we performed PSR staining in ovaries to examine the accumulation of collagen and investigated whether the tendency of collagen deposition differed between *Tinagl1* ^−/−^ and *Tinagl1^flox/flox^* mice during aging. PSR staining (Figure 4A) and the resulting processed color threshold image (Figure 4B) demonstrated intense PSR staining throughout the ovarian stroma in an age‐dependent manner in *Tinagl1^flox/flox^* females. Meanwhile, the appearance of intense PSR staining was delayed to 14‐15 months in *Tinagl1* ^−/−^ females. This timing was consistent with the retarded timing of age‐related decline of ovulation in *Tinagl1* ^−/−^ females. These results suggest that ovarian deposition of collagen is retarded by *Tinagl1* deficiency followed by alleviation of age‐related ovulation decline.

### 3.5 Distribution of Tinagl1 in ovary during aging

**Figure 5:**
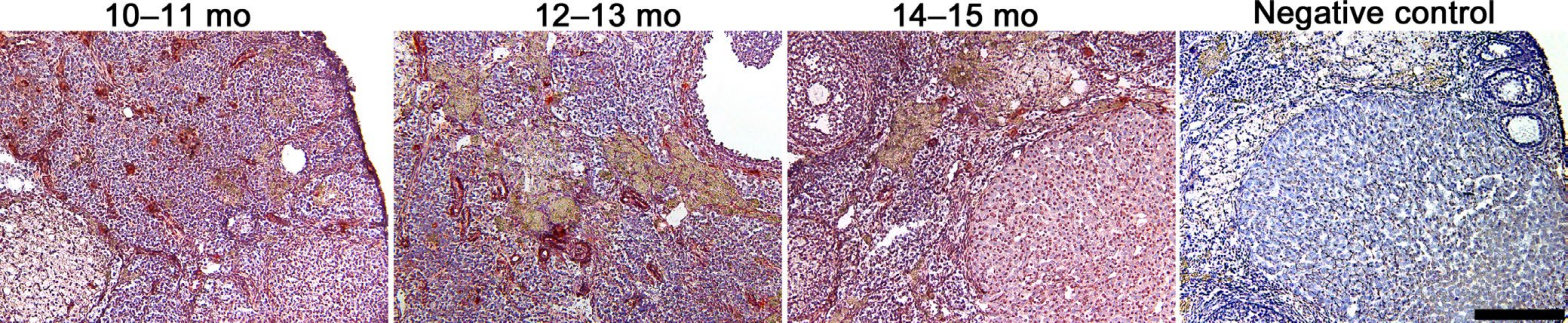
Distribution of Tinagl1 in *Tinagl1^flox/flox^* mouse ovary during aging. The scale bar represents 200 µm

Immunohistochemistry was performed to determine the localization of Tinagl1 expression in *Tinagl1^flox/flox^* female ovary during aging. Tinagl1 protein was expressed in ovarian stroma, blood vessels, theca cells, and corpus lutea (Figure 5). In ovarian stroma, Tinagl1 expression was similar during aging.

## 4 DISCUSSION

Previous work by our group indicated that induced ovulation in 2‐7 months old *Tinagl1^−/−^* mice did not differ compared with *Tinagl1^flox/flox^* mice.^22^ Furthermore, ovulated *Tinagl1*‐null oocytes possessed developmental potential during the preimplantation period.^22^ In contrast, our present study shows that deficiency of *Tinagl1* alleviates the age‐related decline of ovulation, whereas in vitro fertilization followed by embryo culture for ovulated oocytes showed a normal developmental potential for *Tinagl1^−/−^* embryos during the preimplantation period as compared with *Tinagl1^flox/flox^* embryos.

We found that *Tinagl1*‐deficient female mice exhibit alleviation of age‐related ovulation decline. As compared with 8‐ to 9‐month‐old mice, the number of ovulated oocytes from *Tinagl1^flox/flox^* mice decreased in an age‐dependent manner for females more than 10‐11 months old, whereas ovulated oocyte numbers for 8‐ to 9‐month‐old *Tinagl1^−/−^* mice did not differ significantly from those for 10‐ to 11‐ and 12‐ to 13‐month‐old mice but decreased significantly in 14‐ to 15‐month‐old females. PSR staining intensity, indicating collagen deposition, increased in an age‐dependent manner throughout the ovarian stroma in *Tinagl1^flox/flox^* females. Meanwhile, the observation of intense PSR‐stained collagen was delayed to 14‐15 months in *Tinagl1* ^−/−^ females. This timing was consistent with the retarded timing of age‐related decline of ovulation in *Tinagl1* ^−/−^ females. Furthermore, Tinagl1 protein was expressed in ovarian stroma during aging. In mice, reproductive age‐associated fibrosis occurs in the stroma of the ovary. Previous study revealed that PSR staining was minimal in ovaries from reproductively young adult mice and increased in advanced reproductive age, with staining becoming prominent throughout the stroma of aged mice.^9^ Therefore, our current findings suggest that *Tinagl1* deficiency causes delayed reproductive age‐associated ovarian collagen deposition followed by an alleviation of the age‐related decline of ovulation.

Fibrosis occurred in the ovarian stroma with increasing age and increased the inflammatory response in the ovary.^9^ TINAGL1 level was increased in Fabry disease with upregulated inflammation‐related pathways.^27^ Therefore, ovarian expression Tinagl1 may be associated with inflammatory response during ovarian fibrosis. In addition, stiffness of ovary was increased with ovarian stromal fibrosis^28^ and showed that the stiffness around the follicle was associated with follicular development.^29^ Ovarian stromal fibrosis caused the abnormal follicular development at secondary follicle in granulosa cell‐specific *Nrg1* knockout mice and resulted in decreased number of ovulated oocytes.^30^ It has been reported that tissue stiffness and mammographic density are associated with the risk of breast carcinoma development, suggesting that matricellular proteins may influence breast carcinoma prognosis.^31^ Moreover, Tinagl1 suppresses triple‐negative breast cancer progression and metastasis.^32^ Therefore, ovarian Tinagl1 may be associated with ovarian stiffness and depressed follicular development. Recent study reported that TINAGL1 promotes hepatocellular carcinogenesis through the activation of TGF‐β signaling medicated VEGF expression.^33^ Because Tinagl1 is present in blood vessels,^19^, ^21^, ^34^ Tinagl1 also could be related to ovarian blood vessel formation followed by constant cycles of connective tissue remodeling.

Through the processes of ovulation, the ovary undergoes constant cycles of connective tissue remodeling and wound healing, which require a complex interplay between matrix metalloproteinases (MMPs) and tissue inhibitors of metalloproteinases (TIMPs).^35^ TIMPs inhibit the proteolytic activity of MMPs. Therefore, age‐associated changes in the homeostasis of ECM may be associated with an imbalance in the activities of MMPs and TIMPs.^9^ Furthermore, the age‐associated fibrosis could be due to increased synthesis and deposition of collagen or other ECM components and/or altered post‐translational modifications, because α‐smooth muscle actin (α‐SMA), which corresponds to a myofibroblast population,^36^, ^37^ has been observed in the ovarian stroma.^9^ Persistent activation of myofibroblasts can cause excessive fibrotic reactions.^38^ In addition, the study of myofibroblast recruitment and activation in skin wounds using α‐SMA‐GFP transgenic mice demonstrated that Tinagl1 is linked to myofibroblast‐mediated wound healing.^38^ Therefore, it is possible that the alleviation of age‐associated decline of ovulation in *Tinagl1*‐deficient females is caused by reduced connective tissue remodeling during the processes of ovulation.

Under normal physiologic conditions, tissue remodeling in response to injury leads to tissue regeneration without permanent damage. Matricellular proteins are often upregulated after tissue injury^39^ to provide extracellular matrix signals critical for tissue repair processes.^40^, ^41^ These non‐structural ECM proteins are important during embryonic development but are typically restricted to tissue remodeling and wound repair in normal adults.^42^, ^43^, ^44^ Indeed, several studies examining mice deficient in matricellular proteins have identified previously unsuspected consequences stemming from the lack of appropriate interactions between cells and their environment.^10^ Tinagl1 has been identified as a matricellular protein.^19^ In this work, we determined that Tinagl1 plays a role in age‐associated ovulation decline caused by ovarian collagen deposition.

In conclusion, the present study describes new observations including alleviation of age‐associated depression of ovulation caused by retarded ovarian collagen deposition in *Tinagl1*‐null female mice. Although the numbers of ovulated oocytes from *Tinagl1^flox/flox^* mice decreased in an age‐dependent manner in mice more than 10‐11 months old, ovulated oocyte numbers from *Tinagl1^−/−^* mice did not differ significantly among 8‐9, 10‐ to 11‐, and 12‐ to 13‐month‐old females and decreased significantly by 14‐15 months. However, the fertilization potential of ovulated oocytes was not significantly different between *Tinagl1^flox/flox^* and *Tinagl1^−/−^*mice. Furthermore, *Tinagl1* deficiency did not affect the potential of development to the blastocyst stage of ovulated oocytes during the preimplantation period as compared with *Tinagl1^flox/flox^* females. As *Tinagl1* deficiency alleviated the age‐related decline of ovulation, and in vitro fertilization followed by embryo culture for ovulated oocytes showed normal developmental potential during the preimplantation period, investigation of physiologic and/or microenvironmental conditions in the ovary may further elucidate the role of Tinagl1. Such work could yield information that may help to improve assisted reproductive technology, although determination of the definitive role of Tinagl1 in ovary during aging will require further studies involving the ovary‐specific deletion of this gene.

## CONFLICT OF INTEREST

All authors declare no conflict of interest.

## ANIMAL STUDY

All animal experiments described here were approved by the Animal Experimentation Committee at the Utsunomiya University and were performed in accordance with the instructions in the Guide for the Care and Use of Laboratory Animals published by Utsunomiya University.

## HUMAN RIGHTS

This article does not contain any studies with human subjects performed by the any of the authors.

## References

[1] Cui LB , Zhou XY , Zhao ZJ , Li Q , Huang XY , Sun FZ . The Kunming mouse: as a model for age‐related decline in female fertility in human. Zygote. 2013;21:367‐376. 23517725 10.1017/S0967199412000123

[2] Qiao J , Wang ZB , Feng HL , et al. The root of reduced fertility in aged women and possible therapentic options: current status and future perspects. Mol Aspects Med. 2014;38:54‐85. 23796757 10.1016/j.mam.2013.06.001

[3] Faddy MJ , Gosden RG , Edwards RG . Ovarian follicle dynamics in mice: a comparative study of three inbred strains and an F1 hybrid. J Endocrinol. 1983;96:23‐33. 6822780 10.1677/joe.0.0960023

[4] Faddy MJ , Gosden RG , Gougeon A , Richardson SJ , Nelson JF . Accelerated disappearance of ovarian follicles in mid‐life: implications for forecasting menopause. Hum Reprod. 1992;7:1342‐1346. 1291557 10.1093/oxfordjournals.humrep.a137570

[5] Faddy MJ . Follicle dynamics during ovarian ageing. Mol Cell Endocrinol. 2000;163:43‐48. 10963872 10.1016/s0303-7207(99)00238-5

[6] Tarin JJ , Perez‐Albala S , Cano A . Cellular and morphological traits of oocytes retrieved from aging mice after exogenous ovarian stimulation. Biol Reprod. 2001;65:141‐150. 11420234 10.1095/biolreprod65.1.141

[7] Mitsui A , Yoshizawa M , Matsumoto H , Fukui E . Improvement of embryonic development and production of offspring by transferring meiosis‐II chromosomes of senescent mouse oocytes into cytoplasts of young mouse oocytes. J Assist Reprod Genet. 2009;26:35‐39. 19096925 10.1007/s10815-008-9282-6 PMC2649337

[8] Yamada M , Egli D . Genome transfer prevents fragmentation and restores developmental potential of developmentally compromised postovulatory aged mouse oocytes. Stem Cell Reports. 2017;8:576‐588. 28242217 10.1016/j.stemcr.2017.01.020 PMC5355644

[9] Briley SM , Jasti S , McCracken JM , et al. Reproductive age‐associated fibrosis in the stroma of the mammalian ovary. Reproduction. 2016;152:245‐260. 27491879 10.1530/REP-16-0129 PMC4979755

[10] Bornstein P , Sage EH . Matricellular proteins: extracellular modulators of cell function. Curr Opin Cell Biol. 2002;14:608‐616. 12231357 10.1016/s0955-0674(02)00361-7

[11] Syn WK , Choi SS , Liaskou E , et al. Osteopontin is induced by hedgehog pathway activation and promotes fibrosis progression in nonalcoholic steatohepatitis. Hepatology. 2011;53:106‐115. 20967826 10.1002/hep.23998 PMC3025083

[12] Pritchett J , Harvey E , Athwal V , et al. Osteopontin is a novel downstream target of SOX9 with diagnostic implications for progression of liver fibrosis in humans. Hepatology. 2012;56:1108‐1116. 22488688 10.1002/hep.25758 PMC3638324

[13] Syn WK , Agboola KM , Swiderska M , et al. NKT‐associated hedgehog and osteopontin drive fibrogenesis in non‐alcoholic fatty liver disease. Gut. 2012;61:1323‐1329. 22427237 10.1136/gutjnl-2011-301857 PMC3578424

[14] El‐Karef A , Yoshida T , Gabazza EC , et al. Deficiency of tenascin‐C attenuates liver fibrosis in immune‐mediated chronic hepatitis in mice. J Pathol. 2007;211:86‐94. 17121418 10.1002/path.2099

[15] Camino AM , Atorrasagasti C , Maccio D , et al. Adenovirus‐mediated inhibition of SPARC attenuates liver fibrosis in rats. J Gene Med. 2008;10:993‐1004. 18615449 10.1002/jgm.1228

[16] Atorrasagasti C , Aquino JB , Hofman L , et al. SPARC downregulation attenuates the profibrogenic response of hepatic stellate cells induced by TGF‐beta1 and PDGF. Am J Physiol Gastrointest Liver Physiol. 2011;300:G739‐G748. 21311029 10.1152/ajpgi.00316.2010 PMC3094149

[17] Atorrasagasti C , Peixoto E , Aquino JB , et al. Lack of the matricellular protein SPARC (secreted protein, acidic and rich in cysteine) attenuates liver fibrogenesis in mice. PLoS ONE. 2013;8:e54962. 23408952 10.1371/journal.pone.0054962 PMC3569438

[18] Huang Y , Liu W , Xiao H , et al. Matricellular protein periostin contributes to hepatic inflammation and fibrosis. Am J Pathol. 2015;185:786‐797. 25541330 10.1016/j.ajpath.2014.11.002

[19] Li D , Mukai K , Suzuki T , et al. Adrenocortical zonation factor 1 is a novel matricellular protein promoting integrin‐mediated adhesion of adrenocortical and vascular smooth muscle cells. FEBS J. 2007;274:2506‐2522. 17425658 10.1111/j.1742-4658.2007.05786.x

[20] Igarashi T , Tajiri Y , Sakurai M , et al. Tubulointerstitial nephritis antigen‐like 1 is expressed in extraembryonic tissues and interacts with laminin 1 in the Reichert membrane at postimplantation in the mouse. Biol Reprod. 2009;81:948‐955. 19587330 10.1095/biolreprod.109.078162

[21] Tajiri Y , Igarashi T , Li D , et al. Tubulointerstitial nephritis antigen‐like 1 is expressed in the uterus and binds with integrins in decidualized endometrium during postimplantation in mice. Biol Reprod. 2010;82:263‐270. 19776386 10.1095/biolreprod.109.080028

[22] Takahashi A , Rahim A , Takeuchi M , et al. Impaired female fertility in tubulointerstitial antigen‐like 1‐deficient mice. J Reprod Dev. 2016;62:43‐49. 26522507 10.1262/jrd.2015-109 PMC4768111

[23] Takeuchi M , Seki M , Furukawa E , et al. Improvement of implantation potential in mouse blastocysts derived from IVF by combined treatment with prolactin, epidermal growth factor and 4‐hydroxyestradiol. Mol Hum Reprod. 2017;23:557‐570. 28810691 10.1093/molehr/gax035

[24] Junqueira LC , Bignolas G , Brentani RR . Picrosirius staining plus polarization microscopy, a specific method for collagen detection in tissue sections. Histochem J. 1979;11:447‐455. 91593 10.1007/BF01002772

[25] Emde B , Heinen A , Godecke A , Bottermann K . Wheat germ agglutinin staining as a suitable method for detection and quantification of fibrosis in cardiac tissue after myocardial infarction. Eur J Histochem. 2014;58:2448. 25578975 10.4081/ejh.2014.2448 PMC4289847

[26] Matsumoto H , Fukui E , Yoshizawa M , Sato E , Daikoku T . Differential expression of the motin family in the peri‐implantation mouse uterus and their hormonal regulation. J Reprod Dev. 2012;58:649‐653. 22813598 10.1262/jrd.2012-075

[27] Ko Y , Lee C , Moon MH , Hong GR , Cheon CK , Lee JS . Unravelling the mechanism of action of enzyme replacement therapy in Fabry disease. J Hum Genet. 2016;61:143‐149. 26490183 10.1038/jhg.2015.123

[28] Wood CD , Vijayvergia M , Miller FH , et al. Multi‐modal magnetic resonance elastography for noninvasive assessment of ovarian tissue rigidity in vivo. Acta Biomater. 2015;13:295‐300. 25463483 10.1016/j.actbio.2014.11.022 PMC4295766

[29] West ER , Xu M , Woodruff TK , Shea LD . Physical properties of alginate hydrogels and their effects on in vitro follicle development. Biomaterials. 2007;28:4439‐4448. 17643486 10.1016/j.biomaterials.2007.07.001 PMC2034204

[30] Umehara T , Kawai T , Kawashima I , et al. The acceleration of reproductive aging in Nrg1(flox/flox); Cyp19‐Cre female mice. Aging Cell. 2017;16:1288‐1299. 28857490 10.1111/acel.12662 PMC5676068

[31] Fiorino S , Di Saverio S , Leandri P , et al. The role of matricellular proteins and tissue stiffness in breast cancer: a systematic review. Future Oncol. 2018;14:1601‐1627. 29939077 10.2217/fon-2017-0510

[32] Shen M , Jiang YZ , Wei Y , et al. Tinagl1 suppresses triple‐negative breast cancer progression and metastasis by simultaneously inhibiting integrin/FAK and EGFR signaling. Cancer Cell. 2019;35:64‐80.e7. 30612941 10.1016/j.ccell.2018.11.016

[33] Sun L , Dong Z , Gu H , Guo Z , Yu Z . TINAGL1 promotes hepatocellular carcinogenesis through the activation of TGF‐β signaling‐medicated VEGF expression. Cancer Manage Res. 2019;11:767‐775. 10.2147/CMAR.S190390 PMC6339651 30697069

[34] Wex T , Lipyansky A , Bromme NC , Wex H , Guan XQ , Bromme D . TIN‐ag‐RP, a novel catalytically inactive cathepsin B‐related protein with EGF domains, is predominantly expressed in vascular smooth muscle cells. Biochemistry. 2001;40:1350‐1357. 11170462 10.1021/bi002266o

[35] Curry TE Jr , Osteen KG . Cyclic changes in the matrix metalloproteinase system in the ovary and uterus. Biol Reprod. 2001;64:1285‐1296. 11319131 10.1095/biolreprod64.5.1285

[36] Skalli O , Ropraz P , Trzeciak A , Benzonana G , Gillessen D , Gabbiani G . A monoclonal antibody against alpha‐smooth muscle actin: a new probe for smooth muscle differentiation. J Cell Biol. 1986;103:2787‐2796. 3539945 10.1083/jcb.103.6.2787 PMC2114627

[37] Hinz B . Myofibroblasts. Exp Eye Res. 2016;142:56‐70. 26192991 10.1016/j.exer.2015.07.009

[38] Bergmeier V , Etich J , Pitzler L , et al. Identification of a myofibroblast‐specific expression signature in skin wounds. Matrix Biol. 2018;65:59‐74. 28797711 10.1016/j.matbio.2017.07.005

[39] Tracy LE , Minasian RA , Caterson EJ . Extracellular matrix and dermal fibroblast function in the healing wound. Adv Wound Care. 2016;5:119‐136. 10.1089/wound.2014.0561 PMC4779293 26989578

[40] Kyriakides TR , Maclauchlan S . The role of thrombospondins in wound healing, ischemia, and the foreign body reaction. J Cell Commun Signal. 2009;3:215‐225. 19844806 10.1007/s12079-009-0077-z PMC2778594

[41] Murphy‐Ullrich JE , Sage EH . Revisiting the matricellular concept. Matrix Biol. 2014;37:1‐14. 25064829 10.1016/j.matbio.2014.07.005 PMC4379989

[42] Bornstein P . Diversity of function is inherent in matricellular proteins: an appraisal of thrombospondin 1. J Cell Biol. 1995;130:503‐506. 7542656 10.1083/jcb.130.3.503 PMC2120533

[43] Ruan K , Bao S , Ouyang G . The multifaceted role of periostin in tumorigenesis. Cell Mol Life Sci. 2009;66:2219‐2230. 19308325 10.1007/s00018-009-0013-7 PMC11115806

[44] Wu T , Ouyang G . Matricellular proteins: multifaceted extracellular regulators in tumor dormancy. Protein Cell. 2014;5:249‐252. 24563214 10.1007/s13238-014-0023-6 PMC3978162

